# Characterization of Coding Synonymous and Non-Synonymous Variants in *ADAMTS13* Using *Ex Vivo* and *In Silico* Approaches

**DOI:** 10.1371/journal.pone.0038864

**Published:** 2012-06-29

**Authors:** Nathan C. Edwards, Zachary A. Hing, Avital Perry, Adam Blaisdell, David B. Kopelman, Robert Fathke, William Plum, Jordan Newell, Courtni E. Allen, Geetha S., Aaron Shapiro, Chinyere Okunji, Idit Kosti, Noam Shomron, Vahan Grigoryan, Teresa M. Przytycka, Zuben E. Sauna, Raheleh Salari, Yael Mandel-Gutfreund, Anton A. Komar, Chava Kimchi-Sarfaty

**Affiliations:** 1 Laboratory of Hemostasis, Division of Hematology, Center for Biologics Evaluation and Research, Food and Drug Administration, Bethesda, Maryland, United States of America; 2 Department of Biology, Technion-Israel Institute of Technology, Haifa, Israel; 3 Department of Cell and Developmental Biology, Tel Aviv University, Tel Aviv, Israel; 4 National Center for Biotechnology Information, National Library of Medicine, National Institutes of Health, Bethesda, Maryland, United States of America; 5 Center for Gene Regulation in Health and Disease, Department of Biological, Geological and Environmental Sciences, Cleveland State University, Cleveland, Ohio, United States of America; Emory University School of Medicine, United States of America

## Abstract

Synonymous variations, which are defined as codon substitutions that do not change the encoded amino acid, were previously thought to have no effect on the properties of the synthesized protein(s). However, mounting evidence shows that these “silent” variations can have a significant impact on protein expression and function and should no longer be considered “silent”. Here, the effects of six synonymous and six non-synonymous variations, previously found in the gene of ADAMTS13, the von Willebrand Factor (VWF) cleaving hemostatic protease, have been investigated using a variety of approaches. The ADAMTS13 mRNA and protein expression levels, as well as the conformation and activity of the variants have been compared to that of wild-type ADAMTS13. Interestingly, not only the non-synonymous variants but also the synonymous variants have been found to change the protein expression levels, conformation and function. Bioinformatic analysis of *ADAMTS13* mRNA structure, amino acid conservation and codon usage allowed us to establish correlations between mRNA stability, RSCU, and intracellular protein expression. This study demonstrates that variants and more specifically, synonymous variants can have a substantial and definite effect on ADAMTS13 function and that bioinformatic analysis may allow development of predictive tools to identify variants that will have significant effects on the encoded protein.

## Introduction

ADAMTS13 (A Disintegrin-like and Metalloprotease with Thrombospondin type-1 repeats, member-13) plays an integral role in vascular hemostasis by cleaving von Willebrand Factor (VWF) within intact blood vessels under shear stress [1]. At sites of vascular injury, VWF binds to the sub-endothelium and tethers platelets to this site, initiating coagulation. A deficiency in ADAMTS13 activity – either through inactivation by autoantibodies, lack of expression, or a genetic variation affecting function (these primarily include single site codon substitutions) – results in increased VWF thrombogenic potential. Extreme cases of ADAMTS13 deficiency precipitate Thrombotic Thrombocytopenic Purpura (TTP [OMIM 274150] - http://www.omim.org/), a life threatening hematological disease [2].

Single nucleotide polymorphisms (SNPs), originally defined as single site codon substitutions that occur in >1% of the population, are prevalent and are found across the entire human genome coding sequence, with few exceptions. Approximately 962,258 unique SNPs have been reported in the coding sequence of the human genome, although frequency data are not available for all of these SNPs. Therefore, SNPs are now classified as genomic variants and it is no longer possible to distinguish between SNPs and mutations based on their frequency [3]. Mounting evidence suggests that these synonymous (“silent”) variants may impact protein expression and function [4]–[10]. In humans, synonymous variants have been shown to affect mRNA splicing [5], mRNA stability [11] and/or mRNA secondary structure [12]–[15], translation efficiency and kinetics [16], [17], protein folding [10], [18], [19], and protein function [18].

At the inception of this project, we chose to investigate twelve *ADAMTS13* variants – six synonymous variants and six non-synonymous variants (the latter defined as single site codon substitutions that do change the encoded amino acid) and originally listed in the coding region of the gene in the NCBI dbSNP (http://www.ncbi.nlm.nih.gov/snp, last accessed 24 October 2011). Some of these variants have been previously investigated using *in vitro* methods by other researchers [20]–[23]. One hundred and thirty more variants have been added to dbSNP recently, probably as a result of the increased population sequencing from the 1000 Genomes Project. These variants are not subjects of the current study; however, we do plan to include them in future analyses.

Here, we have used a transient expression system to study the effects of the twelve variants mentioned above on mRNA and protein expression levels, protein activity and conformation *ex vivo*, in cells. In addition, we have employed a variety of computational methods to analyze the potential effects of these variants on *ADAMTS13* mRNA splicing, change in mRNA structure, codon usage and amino acid conservation as well as the relationships between the location of these variants in the encoded polypeptide chain and the wild-type (WT) ADAMTS13 (predicted) protein structure.

Substantial differences in protein expression levels, activity and conformation were found between WT ADAMTS13 and ADAMTS13 variants, suggesting that both non-synonymous and synonymous variants in *ADAMTS13* are not neutral. Furthermore, we demonstrate that *in silico* analysis may serve as a tool to identify variants that may potentially have an effect on the protein bearing them, altering its expression levels and/or activity. *In silico* variables with high correlation to *ex vivo* results (Spearman’s rho≥0.6; p-value<0.05) may become important for the characterization of potential TTP patients carrying genetic variants. These *in silico* variables may also be used in the future for developing safer and more effective therapeutic recombinant proteins. This may be achieved by taking into account the predicted effects of variants (and even haplotypes) on ADAMTS13 or any other therapeutic recombinant protein characteristics.

## Results

### Computational Prediction of mRNA Structure/Stability and Analysis of ADAMTS13 mRNA Expression Levels

**Figure 1:**
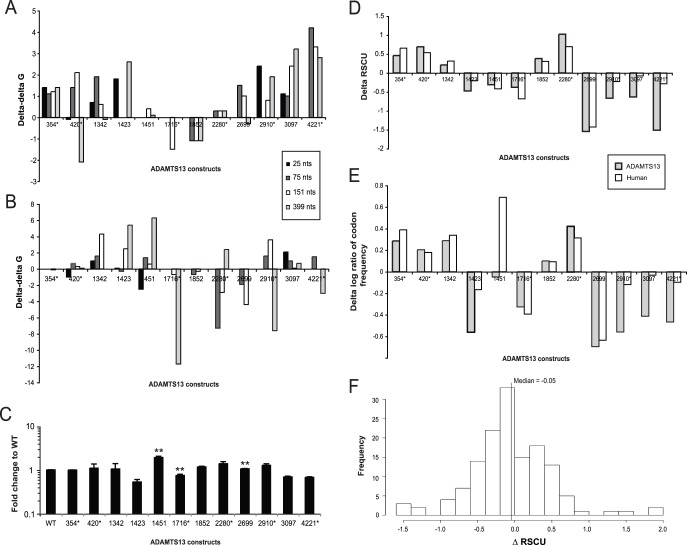
Analysis of mRNA structure/stability, codon usage and expression levels of ADAMTS13 variants: A. mFold ΔΔG values of *ADAMTS13* mRNA fragments: RNA fragments of 25, 75, 151, and 399 nucleotides in length were queried using online mFold server, utilizing default server settings. Variants were centered within the RNA fragments used in the analysis. The most stable structures (lowest ΔG) for both variant and WT *ADAMTS13* variants were chosen. ΔΔG (ΔG variant - ΔG WT) was calculated and is displayed here for each of the variants. **B.** KineFold ΔΔG values calculated using online KineFold server (obtained employing a similar strategy). **C.** *ADAMTS13* mRNA expression levels as determined by qPCR: Analysis of mRNA expression was performed 24 h. post-transfection with quantitative real time PCR on WT *ADAMTS13* and all twelve variants. *GAPDH* was used as a reference and a ΔΔCp was calculated using the average of all WT ΔCp results for comparison. Fold change relative to WT is presented on a logarithmic scale. mRNA expression levels were analyzed in multiple independent transfection experiments utilizing each variant and WT *ADAMTS13* variant, and found to be consistently similar from one experiment to the another (**p-value <0.05). **D.** Differences in Relative Synonymous Codon Usage (RSCU) values between each of the *ADAMTS13* variant codons and the WT codon: ΔRSCU =  Δ(RSCUVariant – RSCUWT); the more positive is the ΔRSCU value, the more common is the variant codon compared to WT codon. RSCU values were calculated using the ADAMTS13 coding region and human codon usage information. **E.** Differences between log ratio of codon usage values for each of the *ADAMTS13* variants and WT. The plotted values are the differences between log ratio of codon usage frequency of the variant and WT codon (Δ(variant – WT)). The more positive the delta, the more commonly used the variant codon is, compared to WT. Values were calculated using the ADAMTS13 coding region and human codon usage information. **F.** Normal distribution of RSCU values for all variants in *ADAMTS13*: The RSCU and log ratio of codon usage values were determined for all variants in the coding region of *ADAMTS13*. MAD scores were assigned to the ΔRSCU and Δlog ratio of all variants. Comparison of MAD scores of twelve variants and all variants in *ADAMTS13* revealed that ΔRSCU and Δlog ratio scores for the twelve variants fell within the distribution of variants in *ADAMTS13*. *ADAMTS13* variants harboring synonymous variants are marked with (*).

Drawing on many previous reports that analyzed the local secondary structure of mRNA, we used mFold [24], a static secondary structure predictor, and KineFold [25], a stochastic secondary structure predictor, to analyze potential changes in the minimum free energy (ΔG) of the mRNA fragments harboring variants under investigation. The ΔΔG (variant ΔG minus WT ΔG) was calculated for mRNA fragments of different lengths (25, 75, 151 and 399 nucleotides), with the variant of interest positioned in the middle of the mRNA fragment analyzed. We have limited our analysis to RNA fragments/lengths described above, as it is currently unfeasible to predict with sufficient accuracy the structure/stability of longer RNA fragments, as the number of possible structures grows exponentially with the sequence length [24]. The absolute ΔΔG values calculated for a given variant by different software programs and for RNA fragments of different lengths as well as their associated trends were not always in agreement with each other, potentially reflecting the exceptional plasticity of RNA, as well as known limitations of the software packages used, *e.g.* only KineFold is able to predict pseudoknots (Figure 1AB). Nevertheless, synonymous variant 354, independently of the length of the RNA fragment analyzed, was predicted (using both programs) to affect RNA stability to a similar extent (although the absolute magnitude of the effects differed for the two programs used), while synonymous variant 420 showed considerably different ΔΔG values and trends associated with RNA fragments of different lengths (Figure 1AB). Overall, the highest ΔΔG calculated by mFold was observed for synonymous variant 4221 (for a fragment of 75 nucleotides in length) and the lowest ΔΔG was observed for synonymous variant 420 (for a fragment of 151 nucleotides in length) (Figure 1A). At the same time, the highest ΔΔG calculated by KineFold was observed for non-synonymous variant 1451 (for a fragment of 399 nucleotides in length) and the lowest ΔΔG was observed for synonymous variant 1716 (for a fragment of 399 nucleotides in length) (Figure 1B). Although, analysis of mRNA structure within the coding region is admittedly a yet-to-be developed research area, the predicted changes in ΔΔGs may lead to change in mRNA stability and/or protein expression levels.

In order to test whether there might be any association between the predicted changes in mRNA stability and mRNA expression levels, we performed qPCR to determine *ADAMTS13* mRNA expression levels in transfected cells. mRNA levels were monitored 24 hours (h.) post-transfection. Significant differences in *ADAMTS13* mRNA levels between cells transfected with a plasmid harboring WT *ADAMTS13* gene and those transfected with *ADAMTS13* variants were observed for variants 1451, 1716, and 2699 (p-value <0.02, 0.02, 0.04 by 2-sided *t* test, respectively) (Figure 1C). On average, the change in mRNA expression levels was not more than 50% of WT (Figure 1C) for the majority of variants under investigation. One of the largest changes, was found for non-synonymous variant 1451, and remained only 191±11% of WT. Additionally, we observed a correlation (Spearman’s rho = −0.62, p-value<0.03) between the mRNA levels (Figure 1C) and their predicted free energies as calculated by KineFold for a nucleotide length of 25 (Figure 1B).

### Computational Analysis of Codon Usage

While several codons may encode a single amino acid due to general degeneracy of the genetic code, not all codons occur at the same frequency throughout the genome and a strong codon bias exists [26]. Moreover, different organisms have distinct codon biases [26] and it is possible that there may be differences in codon biases between tissues within the same organism [27]–[31], reflecting tissue specificity in gene expression. More importantly for the present study, changes in codon frequencies (in addition to the changes in RNA structure) have been shown to affect the local speed of translation and this can also impact co-translational protein folding and result in a protein with different conformation/specific activity [18], [19], [32]. It is believed that the overall impact of a given codon change on the rate of translation and co-translational protein folding would be more detrimental in the case that it would cause a more substantial change in the codon usage frequency [18], [19], [32]. We therefore compared the codon usage frequencies of each individual variant versus WT codon at the same position using two different approaches (see *Materials and Methods* for details). In brief, the differences between a variant’s codon usage and the corresponding WT codon usage were calculated using either the Relative Synonymous Codon Usage (RSCU) values [33] or the log ratio of the human codon usage frequencies and the codon usage frequencies of *ADAMTS13*, and are shown in Figures 1D and 1E, respectively. The twelve variants studied here were chosen at random and the ΔRSCU and Δlog ratio scores of all twelve fall within the distribution of ΔRSCU and Δlog ratio scores of all synonymous and non-synonymous variants in *ADAMTS13* (Figure 1F).

The most substantial change in the log ratio of codon usage frequencies from variant to WT codon as well as the highest ΔRSCU was observed for non-synonymous variant 2699 (Figures 1D and 1E). Three of the largest increases in the Δlog ratio of codon frequency and ΔRSCU, regardless of the analysis method used, are for synonymous variants –354, 420 and 2280– demonstrating that the WT codon in each of the above mentioned cases is rarer than the variant codon. Additionally, five variants revealed different changes in their log ratio of codon frequencies based on genomic and *ADAMT13* codon frequencies: non-synonymous variants 1423, 1451 and 3097 and synonymous variants 2910 and 4221. The most noticeable difference between *ADAMTS13* and genomic codon frequencies was observed for non-synonymous variant 1451 in which *ADAMTS13* absolute Δlog ratio of codon frequency is roughly 16% of the genomic Δlog ratio of codon frequency (Figure 1E), suggesting that the AAA codon (Table 1, see nsSNP 1451) is underrepresented in the ADAMTS13 sequence in comparison with the entire ORFeome.

**Table 1:** ADAMTS13 synonymous and non-synonymous variants investigated in this study.

Base Pairposition	Amino Acid position	Variant ID(based ondbSNP)	Codon ChangeWT -> Variant	AA ChangeWT -> Variant	Domain/Region**	Predicted 2Dstructural elementfrom model	2D element from 3Dfragment (X-ray)	Distance from theend of the nearest2D structuralelement	Length of thenearest 2Delement
354*	118	rs28571612	CCG > CCA	Pro>Pro	Peptidase M12B	T	Not available	−1	11-residue E
420*	140	rs3118667	GCT > GCC	Ala>Ala	Peptidase M12B	S	Not available	−6	13-residue H
1342	448	rs2301612	CAA > GAA	Gln> Glu	Cysteine-rich		H	−3	10-residue H
1423	475	rs11575933	CCA > TCA	Pro> Ser	Cystein-rich		T	3	4-residue E
1451	484	rs28375042	AGA > AAA	Arg> Lys	Cystein-rich		T	1, −2	5-residue H and/or4-residue E
1716*	572	rs3124768	ACA > ACG	Thr> Thr	Spacer		E	4	8-residue E
1852	618	rs28647808	CCC > GCC	Pro> Ala	Spacer		S	1	3-residue E
2280*	760	rs3124767	GGT > GGC	Gly> Gly	TSP type-1 3	E	Not available	3	5-residue E
2699	900	rs685523	GTG > GCG	Val> Ala	TSP type-1 5	E	Not available	0	3-residue E
2910*	970	rs28641026	GTC > GTT	Val> Val	TSP type-1 6	E	Not available	3	6-residue E
3097	1033	rs28503257	GCT > ACT	Ala> Thr	TSP type-1 7	E	Not available	1	4-residue E
4221*	1407	rs1055432	ACC > ACA	Thr> Thr	CUB 2	Not available	Not available	Not available	Not available

* Synonymous ADAMTS13 variants.

** Based on SwissProt annotation of domains and regions.

### Changes in Protein Expression of ADAMTS13

**Figure 2:**
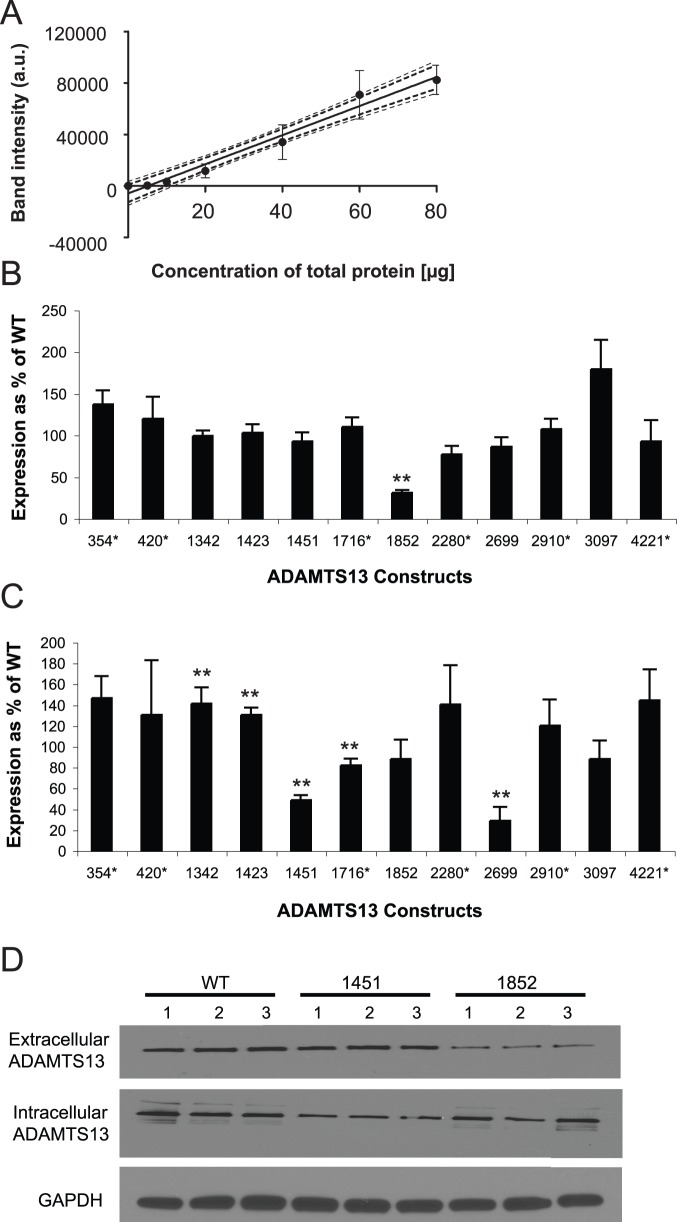
*Ex vivo* analysis of expression levels of ADAMTS13 variants. A. Western blotting data revealing linear dependence between the amount of the loaded protein and the western signal: Media was collected and concentrated 24 h. post-transfection. Increasing amounts of total protein (5, 10, 20, 40, 60 and 80 µg) were subjected to SDS-PAGE analysis. Anti-V5 antibody was used to detect ADAMTS13 and densitometry analysis was performed as described in *Materials and Methods.* The dashed bolded lines indicate 95% confidence limits and the dashed non-bolded lines represent 90% confidence limits. **B.** Extracellular ADAMTS13 Expression (based on immunoblot analysis). Expression results for each variant are presented relative to WT. Multiple independent transfection experiments utilizing each variant and WT ADAMTS13 construct were performed (**p-value <0.05). **C.** Intracellular ADAMTS13 Expression (based on immunoblot analysis): GAPDH and/or β-actin were used as loading controls (not shown). Expression results for each variants are presented relative to WT. Multiple independent transfection experiments utilizing each variant and WT ADAMTS13 construct were performed as above (**p-value <0.05). **D.** SDS-PAGE immunoblot analysis of ADAMTS13 WT, non-synonymous variants 1451 (Arg>Lys) and 1852 (Pro>Ala) expression levels using anti-V5 antibody: Top – extracellular ADAMTS13, middle – intracellular ADAMTS13 and bottom - GAPDH loading control. ADAMTS13 variants harboring synonymous variants are marked with (*).

Immunoblot analysis against the linear V5 epitope (at the C-terminus of ADAMTS13) was used to determine the effect of each variant on the expression of the mutant proteins as compared to the WT ADAMTS13. Both intra- and extra-cellular ADAMTS13 expression levels were determined. Transient transfections with WT *ADAMTS13* construct were performed in triplicate and the resulting protein expression levels were measured at a range of total protein concentrations (loaded on a gel) to ensure that final measurements were done within the region of linear response (Figure 2A). Based on these data, 40 µg of total protein was selected as the optimal loading amount to establish the assay. Our results were linear within 95% confidence limits (Figure 2A). It should also be noted that trends in expression levels of ADAMTS13 constructs harboring the same variant were similar for all experiments done in parallel. Densitometry analyses of the band intensities of WT and variant-containing ADAMTS13 samples were performed and presented as a percent of WT, where WT expression level was set to 100%.

Cells expressing ADAMTS13 non-synonymous variant 3097 revealed the highest increase in extracellular ADAMTS13 expression of 179±35% of WT (p-value = 0.06 by 2-sided *t* test) (Figure 2B). *ADAMTS13* constructs with synonymous variants 354 and 420 showed the second and third highest respective increases in extracellular expression levels as compared to WT (137±17%, p-value<0.06 and 120±26%, of WT, p-value = 0.3 by 2-sided *t* test, respectively). The non-synonymous variant 1852 showed a significant decrease, resulting in less than a third (31±3%, p-value<0.0007 by 2-sided *t* test) of the extracellular WT expression level. As both intracellular and secreted proteins represent the active forms of ADAMTS13 [34], we have also compared the levels of intracellular ADAMTS13 (Figure 2C). The non-synonymous variants 1451 and 2699 and synonymous variant 1716 ADAMTS13 variants showed decreased intracellular expression compared to WT (48±5% p-value<0.003, 29±13% p-value<0.002 and 82±7% of WT p-value = 0.04 by 2-sided *t* test, respectively), while non-synonymous variants 1342 (p-value<0.05 by 2-sided *t* test) and 1423 ADAMTS13 variants (p-value<0.02 by 2-sided *t* test) showed increased (41% and 30%, respectively) intracellular expression compared to WT. In lieu of an increase in extracellular expression above WT, the ADAMTS13 construct harboring synonymous variant 354 also revealed a 146±21% (p-value = 0.06 by 2-sided *t* test) increase in intracellular expression above WT.

ADAMTS13 variants did not show any significant correlation between intra- and extracellular expression (Spearman’s rho = 0.98, p-value>0.7). Mutants highly expressed in the intracellular compartment were not necessarily highly secreted and vice versa. A representative example, in which three independently transfected ADAMTS13 constructs (WT, non-synonymous variants 1451 and 1852) were simultaneously analyzed, is shown in Figure 2D. The ADAMTS13 non-synonymous 1451 variant showed decreased intracellular expression levels compared to WT and unaffected extracellular levels (compared to WT), while the ADAMTS13 non-synonymous variant 1852 variant showed the opposite trend. These results indicate that various ADAMTS13 variants may have different effects on intracellular and extracellular protein expression and these two parameters may not necessarily correlate. These differences in intra- and extracellular expression levels may result from the conformational changes between the ADAMTS13 variants that differentially facilitate or impede their travel through certain intracellular compartments, and/or subject them to enhanced degradation.

We found that changes in intracellular ADAMTS13 expression levels correlated strongly (Spearman’s rho = 0.67, p-value = 0.02) with corresponding changes in RSCU values calculated using human codon frequencies. In addition, changes in mRNA minimum free energy values (ΔΔGs based on KineFold predictions for the 175 nucleotide fragment (Spearman’s rho = 0.69, p-value = 0.012)) unexpectedly correlated with the corresponding changes in ADAMTS13 extracellular expression levels. We found no correlation between *ADAMTS13* mRNA levels and either extracellular protein expression (Spearman’s rho = 0.20, p-value = 0.40) or intracellular protein expression levels (Spearman’s rho = −0.29, p-value = 0.35).

### ADAMTS13 Variants Show Differences in Specific Activity

**Figure 3:**
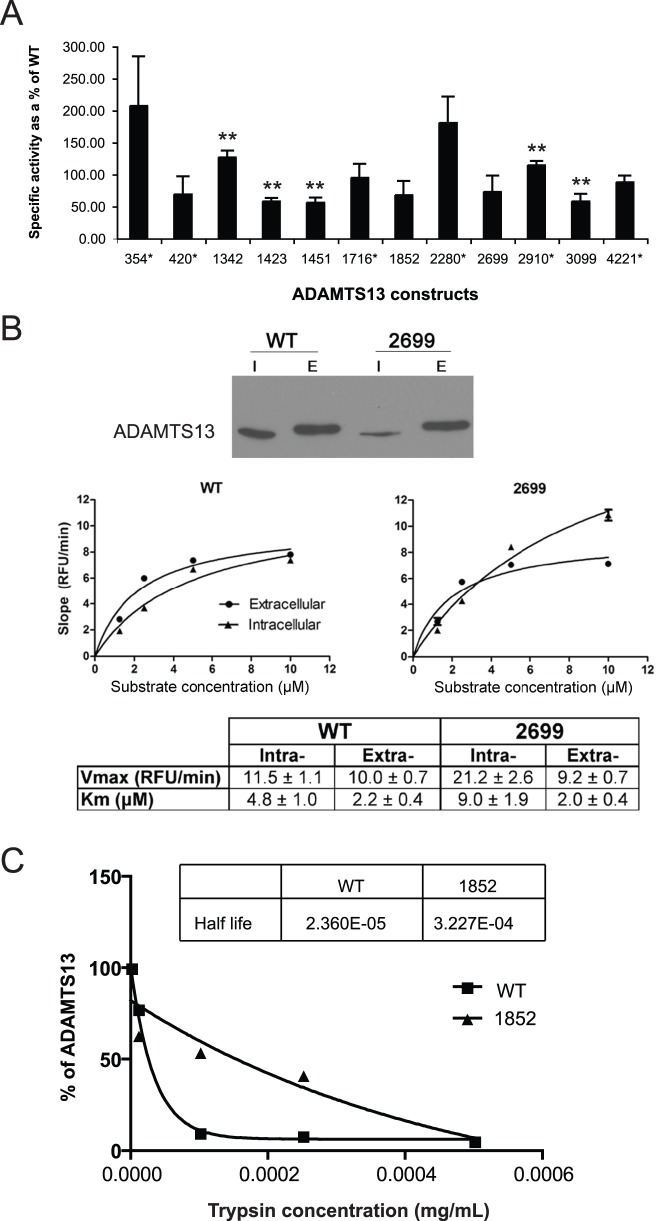
Specific activity of ADAMTS13 variants and limited proteolysis with trypsin. **A.** Specific activity of twelve ADAMTS13 variants vs. WT as determined using FRETS-VWF73 substrate: Samples containing equal amounts of total extracellular protein were incubated with 5 µM of FRETS-VWF73 substrate and fluorescence readings were taken at 5 min intervals over the course of 2 h. Specific activity was calculated as the increase in relative fluorescence units (RFU)/min per unit of ADAMTS13 in the total extracellular protein samples. Extracellular ADAMTS13 protein amounts were determined using immunoblot analysis with anti-V5 antibody as in Figure 2. ADAMTS13 variants harboring synonymous variants are marked with (*) (**p-value <0.05). **B.** Expression and Michaelis-Menten Kinetics of non-synonymous variant 2699 vs. WT protein: HEK293 cells were transfected with WT and non-synonymous variant 2699 and harvest 24 h. post-transfection. Samples containing equal total protein amounts, both intracellular (I) and extracellular (E) (40 µg each), were separated by SDS-PAGE and probed with anti-V5 antibody to determine ADAMTS13 expression. Intracellular (•) and extracellular (▴) protein samples were incubated with 0–10 µM FRETS-VWF73 substrate. Fluorescence released upon cleavage was plotted over time, as previously described, and a best-fit linear regression was calculated to determine the initial rate of the reaction. The change in RFU per unit time was then used to estimate the specific activity of ADAMTS13. Specific activity at each substrate concentration was plotted and a Michaelis-Menten plot was generated using GraphPad Prism software to calculate K_M_ and V_max_ values. **C.** Limited Trypsin digestion of ADAMTS13 variants: Resistance of the intracellular full-length ADAMTS13 non-synonymous variant 1852 (▴) was compared to that of WT (▪) using densitometry analysis of the immunoblot data, as described the *Materials and Methods*. Non-synonymous variant 1852 revealed increase resistance to trypsin digestion compared to WT.

Activity of each ADAMTS13 variant was determined (and compared to that of the WT protein) using the FRETS-VWF73 fluorogenic substrate and normalized to extracellular ADAMTS13 expression levels to yield the specific activity (Figure 3A). Unexpectedly, two ADAMTS13 synonymous variants, 354 and 2280, demonstrated the highest specific activity (207±77% and 181±41%, respectively) compared to WT. Statistical analysis demonstrated that these differences in specific activity were not significant (p-value<0.1 and 0.2 by 2-sided *t* test, respectively). At the same time non-synonymous variants 1451 and 1423 were found to have the lowest specific activity (56±8% with p-value<0.01 and 58±5% with p-value<0.006 by 2-sided *t* test, respectively). We further chose to investigate the non-synonymous 2699 variant (which revealed a ∼25% decrease in specific activity) because the ADAMTS13 protein bearing this variant revealed the lowest intracellular expression level compared to WT protein, with no change in extracellular protein expression levels compared to WT. This suggests possible conformation changes within the mutant protein that perhaps might lead to enhanced intracellular degradation of non-synonymous variant 2699 species that do not pass quality control mechanisms in the ER. However this protein might also travel faster through the secretory pathway and thus the total amount of the extracellular protein may remain unchanged. In order to detect any minor changes in conformation between 2699 and WT, a more detailed kinetic analysis was performed in the case of non-synonymous variant 2699 and WT (Figure 3B, middle panels). V_max_ (RFU min^−1^) and K_M_ (µM) for WT ADAMTS13 and non-synonymous variant 2699 mediated cleavage of the fluorogenic substrate FRETS-VWF73 are shown in Figure 3B. These data revealed an approximately 2-fold difference estimated in K_m_ and V_max_ values between the intracellular forms of the non-synonymous variant 2699 and WT proteins, respectively. Thus, our analysis of the specific activity of 12 ADAMTS13 variants and the detailed kinetic analysis of non-synonymous variant 2699 clearly demonstrated differences in specific activity as compared to WT.

### Limited Trypsin Digestion Suggests that ADAMTS13 Variants may have Different Conformations

To probe and compare the conformations of the variant ADAMTS13 proteins, we subjected them to limited proteolysis with trypsin. We specifically chose to investigate non-synonymous variant 1852, because this ADAMTS13 variant revealed the lowest extracellular expression levels and reduced specific activity as compared to the WT protein. We hypothesized that the differences in expression and function may result from conformational changes within the protein. Increased resistance to limited proteolysis with trypsin was revealed for non-synonymous variant 1852, as compared to WT (Figure 3C).

### Analysis of ADAMTS13 Structure and Locations of Variants

**Figure 4:**
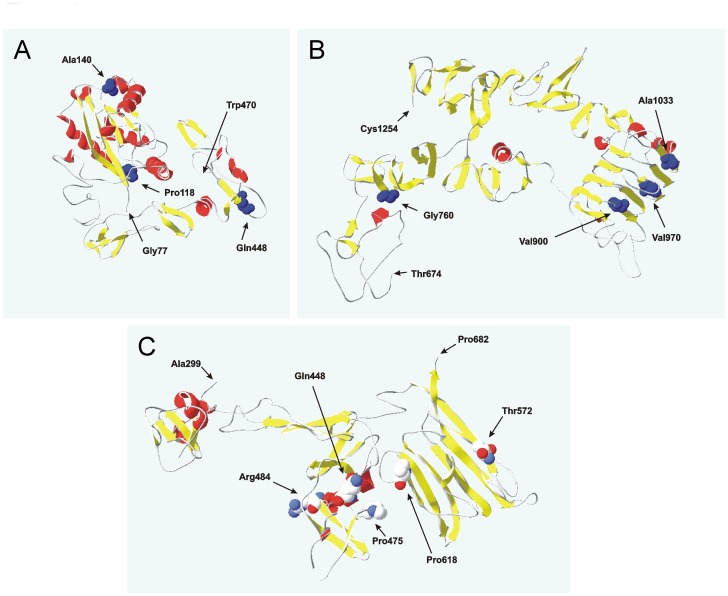
Ribbon diagrams of the ADAMTS13 fragments. Two upper panels: Left - comprising residues (77–470) and right (674–1254) built using comparative modeling (3D-PSSM algorithm). Bottom panel ADAMTS13 X-ray structure residues (287–682) PDB ID 3GHM. Helices are depicted in red and beta-structures are in yellow. Residues encoded by codons/variants under investigation are denoted with residue number (van der Waals’ radii of the side chains are shown). Beginning and end of each fragment are also denoted with a residue number.

It has been suggested that not every non-synonymous or synonymous codon substitution would have an equal impact on protein folding in the cell [17]. The impact of any given variants on the folding of any given protein was suggested to be dependent on the effects produced by variants on the stability and structure of the folding intermediates forming along the co-translational folding pathway [17]. Substitutions that affect codons encoding structurally important residues and/or protein fragments (*e.g.* domain linkers) were suggested to elicit more substantial effects on protein folding in comparison with other non-synonymous or synonymous variants [17]. Therefore, we have attempted to predict and analyze the structure of the ADAMTS13 protein and locate the residues encoded by codons/variants under investigation onto the predicted structure (Figure 4, Table 1). Although prediction of the protein structure is yet a very challenging task and cannot be considered proof that the predicted structures will form, we believe that this prediction can be taken into account with certain level of precaution. Comparative modeling of the ADAMTS13 structure using the 3D-PSSM algorithm [35] yielded two protein fragments (comprising residues 77–470 and 674–1254 respectively) and allowed us to conclude that variants under investigation might affect residues located at the edges of the secondary structures (most likely beta-structures) (Figure 4, top two panels; Table 1). It should also be noted that the structure of the ADAMTS13 fragment (comprising residues 287–682) has been recently solved by X-ray crystallography (PDB ID 3GHM) and thus we were able to extend our analysis onto this fragment (which was not covered by *in silico* predictions). Similar to *in silico* findings, 4 out 5 variants in the ADAMTS13 (287–682) X-ray fragment encode residues located in beta-structures (Pro475 (ADAMTS13 variant 1423), Arg484 (ADAMTS13 variant 1451), Thr572 (ADAMTS13 variant 1716) and Pro618 (ADAMTS13 variant 1852), respectively) with 2 (ADAMTS13 variants 1451 and 1852) located at the very edges of the secondary structures (Figure 4, bottom panel; Table 1). This observation complements our *in silico* predictions.

### Analysis of Conservation of Residues Associated with ADAMTS13 Variants

An interesting question to consider is whether ADAMTS13 variants under investigation are found at codons that encode in WT ADAMTS13 evolutionary conserved amino acid residues. One would expect that non-synonymous variants will be found at less conserved residues, as variants at conserved residues are expected to severely affect protein function, while synonymous variants, should, in principal, be subjected to less evolutionary pressure. To answer this question, we have determined the conservation of the ADAMTS13 amino acid residues using sequence alignments of 50 homologues proteins from Swissprot database (http://www.uniprot.org/, last accessed 7 June 2011) and by employing the ConSeq server [36]. The ConSeq server scores the conservation of amino acids from 1 (not conserved) to 9 (most conserved). Overall, as shown in Table 2, the conservation of the 12 residues under investigation was relatively low, albeit on average ranging from not conserved to moderately conserved (Table 2). The highest conservation was observed for Thr1407 associated with synonymous variant 4221. Notably, we did not observe a substantial difference in the conservation values between synonymous and non-synonymous variants.

**Table 2:** Conservation of ADAMTS13 variants across species.

Base-pair position	Amino-acid position	Conservation score
354*	118	6
420*	140	3
1342	448	4
1423	475	5
1451	484	4
1716*	572	5
1852	618	1
2280*	760	3
2699	900	5
2910*	970	1
3097	1033	6
4221*	1407	7

* Synonymous ADAMTS13 variants.

Conservation scores were calculated using tools from the ConSeq server (http://conseq.tau.ac.il/ver1.1/index.html). The conservation ranges from 1 (not conserved) to 9 (most conserved).

## Discussion

To our knowledge this is the first study that simultaneously analyzes a large number of *ADAMTS13* variants by employing a variety of experimental approaches and *in silico* tools. Our *ex vivo* results revealing differences in ADAMT13 expression levels and activities are fully in agreement with the range of protein activities observed in healthy individuals (A summary of *ex vivo* results can be found in Table S2). For example, Bohm and colleagues [37] reported similar changes in ADAMTS13 activities up to a 135% increase or 50% decrease in ADAMTS13 activity, using VWFcp as measured by the RCo-based assay, and similarly, Zhou and Tsai [38] reported that ADAMTS13 activity may range from 70% to 170% in normal individuals. At the same time, Mannucci and colleagues [39] demonstrated that the expression levels of ADAMTS13 in individuals of O blood type or non-O blood type, range from ∼35% to ∼170%, respectively. However, one should note that the exact activity level of each ADAMTS13 variant observed in *ex vivo* experiments might not precisely correlate with its activity in plasma. A single variant should not be compared to a variant coupled with other variants that often appear together as a frequent haplotype, which may have an alternative significant effect on hemostasis. Information regarding the frequency of variants in various populations is not very abundant and the present study cannot accurately mimic natural ADAMTS13 haplotypes without obtaining additional data that will ultimately require sequencing of a multitude of patient populations. However, we believe that the obtained information may yet be very useful in assessing the effects of individual variants.

Reports in the last five years have indicated that not only non-synonymous variants, but also synonymous ones, can have substantial effects on protein expression and function [5], [8], [40]. Traditionally, recombinant protein therapies and gene therapeutics have introduced variants (most commonly synonymous variants) in order to facilitate increased protein expression [41]. Similarly, the DNA sequence used in development of recombinant protein therapeutics sometimes contains one or more variants and little attention has been previously paid to the effects of such genetic variants. We believe the results of the present study as well as the tools and approaches that we have employed and developed for the analysis of ADAMTS13 variants may be helpful in analyses of the effects of variants found in other genes/proteins.

The results presented here clearly demonstrate that both non-synonymous and synonymous variants can affect protein expression levels and function (Figures 2B, 2C, and 3A). While highlighting the effects of non-synonymous variants on ADAMTS13 conformation and activity (Figures 3B and 3C), our study strongly suggests that the effects of neither non-synonymous nor synonymous variants should be neglected in any gene sequence. Importantly, nine of the twelve *ADAMTS13* variants under study didn’t have any substantial effect on mRNA expression levels (Figure 1C), thus suggesting that their effects on protein expression and function were at the post-transcriptional level. For the constructs that demonstrated significant differences in mRNA expression levels, i.e. 1451, 1716, and 2699, we found no corresponding change in extracellular protein expression levels or specific protein activity. mRNA expression levels of non-synonymous variants 1451 and 2699 were significantly higher than that of WT, but this could not account for the differences observed in protein expression levels, as intracellular protein expression levels of both variants were found to be significantly lower than WT and no change in extracellular expression, compared to WT was observed. Synonymous variant 1716 exhibited significantly lower mRNA expression levels compared to WT, and this difference may account for significantly lower intracellular protein expression levels of this variant compared to WT. However, at the same time no corresponding change in extracellular protein expression levels was observed for this synonymous variant as compared to WT. These findings are consistent with previous reports indicating that some variants may affect mRNA expression levels [42]. Further, analysis of the changes in mRNA expression levels and intra- or extracellular protein expression levels and specific activity revealed no significant correlation. It is well known that in higher eukaryotes only a small fraction of the changes at the proteome level is directly reflected by the changes at the transcriptome level [43] thus highlighting an important role played by the translation and post-translational controls of gene expression. Yet, the possibility that changes in protein expression levels observed in the present study may also arise as a result of alternative mRNA splicing [34] could not be completely ruled out. However, putative alternative splicing would not affect mRNA levels, but might impact efficiency of translation. Computational analysis done in the present study using a variety of approaches [44]–[47] indicated that this scenario is very unlikely. No novel splice sites were predicted to arise in *ADAMTS13* mRNA due to variants under investigation (Table S1).

Therefore, the reported changes in protein expression levels are very likely to arise at the post-transcriptional level. Changes in mRNA structure/folding and minimum free energy may represent one of the reasons for altered protein production. Interestingly, we found a negative correlation between the ΔΔG values of the mRNA 175-nucleotide fragments harboring *ADAMTS13* variants (when calculated using KineFold, Figure 1B) and extracellular ADAMT13 protein expression levels (Figure 2B). This negative correlation is rather unexpected, since one would normally expect the opposite–RNA structures with higher minimum free energies are expected to impede efficient protein translation, while lower minimum free energies are predicted to facilitate it. While these results may yet suggest that changes in the predicted minimum free energy of mRNA within the coding sequence may reflect protein expression levels, they may also underline the importance of post-translational events in the secretory pathway that alter the amount of the final secreted product. It should also be mentioned that eukaryotic mRNAs do not exist in cells as naked polynucleotides, but rather are represented by messenger ribonucleoprotein complexes mRNPs [48]. It was shown that mRNA binding proteins may affect almost every aspect of mRNP metabolism from transport to localization, translation and turnover [48]. Variants may produce changes in the mRNA sequence/structure affecting its association with specific proteins and thus altering the fate of the encode protein. However, such effects are extremely difficult to predict. It is important to note that we did not observe any correlation between ΔΔG mRNA values and the changes in intracellular protein expression levels (Figure 2C) (Spearman’s rho = 0.02, p-value = 0.96), further suggesting that the observed relationship between changes in mRNA minimum free energy and extracellular protein expression is likely due to the secondary effects perhaps related to the altered protein conformation and stability rather than to the direct effects related to the efficiency of protein translation.

It should be noted however that our analysis of mRNA structure and stability has a number of drawbacks and is limited by the centering of the variant nucleotide position within the queried nucleotide sequence. Previously, De Smit and van Duin quantitatively demonstrated the relationship between translational efficiency and mRNA structure at the initiation site; an increase in the stability corresponds to a decrease in initiation rate, which was measured by expression levels of RNA bacteriophage MS2 gene [49]. This observation was further supported in a study utilizing a synthetic library of 154 GFP gene variants expressed in *E. coli* that varied randomly at synonymous sites [50] and more recently in a genome wide association study in yeast [51]. However, in prokaryotes and lower eukaryotes mRNPs are less abundant and thus the impact of the effects at the polynucleotide level can be better seen and/or be more pronounced [48]. It should be also mentioned that it has been suggested that after translational initiation (particularly in higher eukaryotes), the ribosome can, in most cases, locally destabilize secondary structures and move along the message without any significant delays [52], yet complex and stable structures close to the initation site may be detrimental to elongation process [11]. Overall, GC content of the mRNA, which may impact the stability of the mRNA structure, was also reported to alter protein expression levels in mammalian cells as measured using a GFP reporter system [53]. In addition, Bartoszewski and colleagues recently reported that a synonymous variant (in position 507) in the coding region of the human *CFTR* may alter mRNA structure and reduce translation rate and expression of CFTR, as compared to WT protein in addition to the known variant in position 508 [54]. While the data at present are insufficient to draw a conclusion with regard to the predicted structure of mRNA and protein expression levels [55], our results suggest that analysis of the changes in mRNA free energies associated with variants of interest bears further investigation which may lead to their development as predictive tools for protein expression levels.

To look at other possibilities we decided to explore a possible relationship between ADAMTS13 expression levels and changes in codon usage, a known mediator of protein expression, associated with variants under investigation. We found a strong correlation between intracellular ADAMTS13 levels and the changes in codon usage as measured by RSCU (Figure 1D). This strong correlation between *in vitro* and *in silico* data may enable us to establish a number of predictive tools for future analysis of other variants and their effects. However, more data would be required in order to make such type of predictions truly reliable.

We further chose to look at ADAMT13 specific activity as a measure of protein folding/conformation (Figure 3A). Fluorogenic FRETS-VWF73 substrate [56], [57] was chosen to test ADAMTS13 activity due to the following reasons: (i) It has been reported in an international independent blind study that FRETS-VWF73 is the most reproducible ADAMTS13 activity assay [58]. (ii) Data from Tripodi *et al.* suggests that inter-individual variation between normal subjects is more pronounced during shorter incubation times: therefore, initial rate of the specific activity by FRETS-VWF73 was used here as a means of measuring ADAMTS13 activity [59]. In choosing the FRETS-VWF73 assay we are not accounting for possible *in vivo* interactions between ADAMTS13 variants and the full-length VWF. By eliminating the complexity of confounding variables, such as substrate binding affinity and cleavage, the ADAMTS13-mediated proteolysis could be directly quantified for all twelve variants and WT.

Analysis of specific activity of non-synonymous 2699 ADAMTS13 variant vs. WT revealed significant differences in K_M_ and V_MAX_ between the intracellular forms but no difference between the extracellular forms (Figure 3B). Our hypothesis is that intracellular non-synonymous variant 2699 consists of several forms, some of them with conformation(s) that allow higher affinity to the substrate and hence activity, but they could not pass the ER quality control to be secreted [60]–[64]. Upon secretion, the WT and the variant carry similar activity. Moreover, the observed increases and decreases in activity of the polymorphic ADAMTS13 variants examined in this study were quite interesting considering the fact that these variants do not reside in the active site. Therefore, the effects of these variants on the function of ADAMTS13 may be exerted by way of altered conformation between variants and WT. Limited trypsin digestion demonstrate that the WT and variants have different trypsin sensitivity possibly due to exposure of different trypsin sites *e.g.* the non-synonymous 1852 variant showed reduced susceptibility to trypsin as compared to WT protein (Figure 3C).

It is also interesting to note that many variants under discussion are predicted to occur in beta-structures/their edges (Figure 4, Table 1). In particular, non-synonymous variant 1852 results in a substitution of Pro to Ala at the edge of a short 3-residue beta-structure which is a part of an extended antiparallel beta-sheet, forming beta-sheet sandwich (Figure 4). Beta-sheets, as a rule, are thermodynamically more stable than alpha-helices and hence their impact on overall protein folding/stability is greater. Therefore, variants in beta-sheets are expected to produce greater impact on protein folding [65]. Our analysis however didn’t reveal any statistically significant association between the location of variants and the residue conservation (Table 2). The exact reasons why synonymous substitution of CCG to CCA encoding Pro118Pro (synonymous variant 354) results in a substantial increase of ADAMTS13 specific activity, while substitution of GGT to GGC encoding Gly760Gly (synonymous variant 2280) did not, remain unclear. Both synonymous variants Pro118Pro (variant 354) and Gly760Gly (2280) were predicted to be located at the edges of the beta-structures. Perhaps, CCG to CCA substitution may facilitate cis-trans Pro118 isomerization and thus lead to an increased ADAMTS13 specific activity.

In sum, we have demonstrated that non-synonymous and synonymous variants can significantly alter both intracellular and extracellular protein expression as well as protein activity. Currently, there are only a handful of *ex vivo* studies focused on ADAMTS13 variants, however to our knowledge there have been no comprehensive reports detailing such an extensive array of variants for mRNA and protein expression as well as protein specific activity. Our results concur with a previous report by Plaimauer *et al.* establishing that the expression of intracellular non-synonymous variant 1852 is similar to WT but extracellular expression is only 27% of WT (we report 28% of WT) [21]. Plaimauer *et al.* also reported the extracellular expression of non-synonymous variant 1342 to be 95% of WT; similarly Kokame *et al.* reported similar expression of non-synonymous variant 1342 as compared to WT (although careful quantitation of the products was not performed in this study) [20], [21]. However, we found a slightly larger decrease in the expression of ns1342 ADAMTS13 variant as compared to WT. Furthermore, Kokame *et al.*, observed that non-synonymous variant 1423 showed no change in expression as compared to WT [20]. However, we detected a 12% increase in extracellular expression as compared to WT for non-synonymous variant 1423.

Other reports have investigated several of the *ADAMTS13* variants studied here alone or in concert with additional variants, however their results cannot be directly compared to the results reported here due to differences in the expression systems and methods used; yet, some valuable information can be extracted. In a different system which examines the expression and function 72 h. post-transduction, Tao *et al.* used a synonymous 3097 ADAMTS13 variant as a positive control and observed equal extracellular expression and VWF-cleavage activity compared to WT ADAMTS13 in HeLa cells [23]. Interestingly, at the same time, secreted WT ADAMTS13 was not expressed at levels comparable to that of the synonymous variant 3097 during short-term expression which might reflect the situation of clot formation *in vivo*. In addition to these *ex vivo* studies, Schettert *et al.* reports an association between the non-synonymous variant 2699 and increased risk of death by an adverse cardiovascular event and lower cholesterol levels, while no such association was found for the 1852 variant [22]. The correlation between cardiovascular events and expression and specific activity of individual variants has yet to be determined. Therefore, the significant decrease in expression of the non-synonymous variant 1852 and a lack of adverse cardiovascular events in patients may suggest other complex factors.

So, how do variants under study, particularly synonymous variants, exert their effects (leading to altered protein conformation) without altering protein composition? One of the elements that might influence protein folding is codon-usage bias. A co-translational, sequential folding model which incorporates a folding funnel characterized by conformation intermediates and an eventual free energy minima ground-state conformation has been suggested as a plausible hypothesis for *in vivo* protein folding [10]. Recent evidence has demonstrated that codon usage can fine-tune this co-translational folding as the ribosome pauses at rare codons or speeds up at common codons providing a delay or lack thereof in the growth of the nascent polypeptide [19]. We hypothesize that the conformational differences observed between synonymous *ADAMTS13* variants may result from the changes in codon usage and corresponding ribosomal translation speeds. This change in translation speed prompts a divergence from the WT folding pathway and therefore might create a new protein conformation. Statistical analysis revealed no inherent bias in our codon usage tools (RSCU and log ratio) resulting from our choice of twelve variants, compared to the RSCU and log ratio of all variants within ADAMTS13. No correlation was found between the ΔRSCU using genomic codon usages and the specific activity of ADAMTS13 using the FRETS-VWF73 substrate. Extracellular protein expression may be significantly influenced by variant-induced conformational changes and this may explain an additional lack of correlations between specific activity and *in silico* analyses. This indeed suggests that codon usage plays an important role in the protein activity implying that codon usage affects the local rates of translation and thus protein folding and specific activity.

In the last few years, the general belief that synonymous variants are silent has been reevaluated due to the accumulation of data suggesting that they can interfere with the mRNA structure, codon usage and/or conformation of the resultant protein, thereby possibly affecting its function. Moreover, many recombinant proteins carry variants or common haplotypes with currently little attention being paid to which haplotype is used in their production. A set of experimental approaches and tests used here are precise enough to distinguish between slight changes in protein expression and activity levels. Therefore, we believe that these tests and type of analysis may serve as useful tools to better understand the effects of different variants in any target protein and may be also used during the design and evaluation of therapeutic recombinant proteins.

## Materials and Methods

### Cell Lines, Culture and Transfection Conditions

Human embryonic kidney cells (HEK293– ATCC; Manassas, VA) were grown in Dulbecco’s Modified Eagle Medium (DMEM) (Quality Biological, Inc; Gaithersburg, MD) with 1% glutamine (Quality Biological, Inc), 1% penicillin- streptomycin (Hyclone; Logan, UT) and 10% fetal bovine serum (Quality Biological, Inc) at 37°C under humid conditions in 5% CO_2_. 1.2×10^6^ cells were plated 24 hours (h.) before transfection in 2 mL growth medium in 6-well plates. Prior to transfection, DMEM was replaced with 1.6 mL Opti-MEM reduced Serum Medium (Invitrogen; Carlsbad, CA). Cells were transfected with 1 µg of plasmid DNA using 6 µL of Lipofectamine 2000 (Invitrogen) in a total of 400 µL Opti-MEM (Invitrogen). Independent transfections using the variant and WT constructs were performed in triplicate for each variant. A ratio of 1∶9 and 1∶5 eGFP: ADAMTS13 was used with the same transfection conditions for co-transfection of eGFP and ADAMTS13 in order to confirm similar transfection efficiencies of the WT ADAMTS13 and each of the variants (data not shown).

### Plasmids and Site Directed Mutagenesis

*pcDNA4-ADAMTS13* (a gift from Dr. Evan Sadler; St. Louis, MO), which carried a full-length *ADAMTS13* (NM_139025) cDNA conjugated to V5 and poly (His) tags was used as a backbone for all constructs. Site directed mutagenesis (SDM) was performed using the QuikChange Site Directed Mutagenesis Kit (Stratagene; Cedar Creek, TX) to change this sequence to WT. This sequence was then used as the WT reference standard and as the backbone for all twelve variants containing constructs produced via SDM. Bi-directional sequencing was used to confirm that variants were successfully introduced. An empty vector was used for mock-transfection control studies. All *ADAMTS13* plasmids were purified using cesium chloride gradient (performed by Lofstrand Labs Limited; Gaithersburg, MD) to obtain high quality supercoiled plasmids. Plasmid expressing enhanced green fluorescence protein (Clontech peGFP-C1, referred here as eGFP) was used for co-transfection assays. All the variants investigated in this study are described in Table 1.

### Cell Harvest: Cell Lysate and Media Preparation

To detect minor changes in ADAMTS13 expression or function, we chose to harvest cells 24 h. post–transfection because ADAMTS13 expression and function are in a linear response range. Cells and media were collected, cell lysates were prepared and cell media was concentrated, as previously described [18], [66]. Protein concentration was measured using Bio-Rad Protein Assay according to the manufacturer’s instructions (Bio-Rad; New York, NY) or by A280 using a NanoDrop 2000C (Thermo Scientific; Wilmington, DE). Concentrated media and cell lysates were aliquoted and stored at −80°C until further use. The following controls were applied in order to assure similar condition between different transfection experiments: (i) Only high quality, supercoiled DNA plasmids were used for transfections, (ii) eGFP co-transfections were carried out and flow cytometry was used to determine equal transfection efficiencies, as specified below, (iii) the exact same number of cells were plated for each transfection, (iv) each study included a transfected WT plasmid for internal comparison among variants transfected concurrently, and (v) each study included a transfected empty plasmid, a negative control which was used for expression and function studies.

### RNA and qPCR Preparation

RNA was isolated (24 h. post-transfection) using RNeasy Plus Mini Kit (Qiagen Sciences, Germantown, MD) following manufacturer’s instructions. Reverse Transcription was carried out as previously described [34]. Quantitative real time PCR (qPCR) was done using the LightCycler RNA master SYBR Green Kit and LightCycler 480 (Roche Applied Science, Indianapolis, IN), using 1 ng of total RNA with an initial melting cycle of 30 seconds (s) at 95°C followed by 40 cycles of 8 s at 95°C, 12 s at 58°C and 10 s at 72°C. *ADAMTS13* was probed with 5′-tcacagccaacctcacctcg’3′ (Forward (F)) and 5′-ccgcacctgccggttac-3′ (Reverse (R)) primers using *GAPDH* as a reference gene with primers 5′-tcgtggagtccactggcgtctt-3′ (F) and 5′-tggcagtgatggcatggactgt-3′ (R). Crossing Point (Cp) values were obtained and presented in the ΔΔCp method using WT as the comparative control for each variant as previously described [67].

### SDS-PAGE Immunoblotting

Samples were sonicated twice for 5 min., boiled in SDS reducing buffer for 10 min. and then resolved by electrophoresis in NuPAGE 7% Tris-Acetate gels (Invitrogen). Mouse anti-V5 primary antibody (Invitrogen) and goat anti-mouse HRP secondary antibody (Thermo Scientific) were used to detect the transfected ADAMTS13. Antibody staining was detected using Amersham ECL Western Blotting Detection Reagents (GE Healthcare; Piscataway, NJ) on Hyblot CL Autoradiography film (Denville Scientific, Inc.; Metuchen, NJ). Densitometry analysis of band intensities was performed using ImageJ 1.42 q software (http://rsbweb.nih.gov/ij/) or the 4000 MM Pro Image Station (CareStream Health, Inc; Woodbridge, CT) with CareStream software. The following controls, in addition to those specified under *cell harvest: cell lysate and media preparation* subsection, were applied in order to assure reproducibility between different transfection experiments and exclude results due to method variations: (i) samples for Western blotting were loaded per total protein concentrations, (ii) a loading control to verify equal total protein loading using anti-β-actin (Abcam, Inc.; Cambridge, MA) or anti-GAPDH (Santa Cruz Biotechnologies; Santa Cruz, CA) were used in lysate samples, (iii) purified recombinant ADAMTS13 (rADAMTS13 - a gift from Dr. Friedrich Scheiflinger of Baxter Innovations GmbH; Vienna, Austria) was used as a control, (iv) immunoblot analysis of three independent transfections using the linear V5 epitope, was used to determine the internal variation of the variants on the level of ADAMTS13. eGFP co-transfections were performed and harvested 24 h. post-transfection as described above. Cells were washed twice with PBS and eGFP was detected in FL-1 to determine transfection efficiencies between ADAMTS13 plasmid constructs that were shown to be similar (e.g. the median arbitrary fluorescence units of the empty pcDNA3.1 plasmid and synonymous variant 420 was 524.2 and 556.9, respectively).

### Trypsin Digestion

ADAMTS13-cell lysates (1852 vs. WT) were diluted using 100 mM Tris-HCl (Fisher Scientific; Fair Lawn, NJ) and subjected to trypsin digestion for 5 minutes (min.) at 37°C with increasing concentrations of freshly made enzyme (Sigma-Aldrich; St. Louis, MO) at the following concentrations ranges 0–0.0005 mg/mL. The reaction was stopped with 1 mg/mL Trypsin soybean inhibitor (Sigma-Aldrich) and analyzed by immunoblotting as described below. Half life of the protein was calculated using GraphPad Prism software.

### FRETS-VWF73 Activity Assay

Fluorogenic FRETS-VWF73 substrate (Peptides International; Louisville, KY) was prepared and assayed according to manufacturer’s instructions and as previously described [66]. Fluorescence readings were taken at 3–5 min. intervals for 2 h. at 30°C by the Infinite F5000 spectrophotometer (Tecan US; Durham, NC). Equal total volume of media was assayed and normalized according to immunoblot densitometry data. FRETS-VWF73 was analyzed using the rate of ADAMTS13 activity as a change in fluorescence per unit of protein per min. An expanded activity assay was performed for the non-synonymous variant 2699 using a range of FRETS-VWF73 substrate concentrations, 0, 2.5, 5.0 and 10 µM. The kinetics of ADAMTS13 protease activity were obtained by plotting this specific activity of the enzyme as a function of substrate concentration and a Michaelis-Menten plot was generated using GraphPad Prism software.

### Relative Synonymous Codon Usage

Relative Synonymous Codon Usage (RSCU) was calculated for all variants in the coding region of *ADAMTS13* as described by *Sharp and Li*

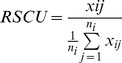
where x_ij_ is the number of occurrences of the j^th^ codon for the i^th^ amino acid, which is encoded by n_i_ synonymous codons [33], [68]. Each variant was analyzed relative to both the codon frequency within *ADAMTS13* cDNA as well as the codon frequency within the entire human genome using the Codon Usage Database (http://www.kazusa.or.jp/codon/, last accessed 21 May 2010) [69]. The *ADAMTS13* constructs used here are under transcriptional control of a CMV promoter, which was reported to have a 2,500% increase in activity when compared to a human BL-3 promoter [70]. Comparison of the viral promoters SV40, EMC, HBV and CMV (which all carry high activity compared to human promoters) in an adeno-associated viral infection system revealed significantly higher expression of Blood Coagulation Factor IX under a CMV promoter [71]. Therefore, the massive increase in ADAMTS13 under the CMV promoter compared to basal ADAMTS13 expression levels would alter the various natural tRNA concentrations within the cell by depleting more quickly tRNAs that bind to highly used codons in *ADAMTS13*. Interestingly, the CG content of the coding region of WT *ADAMTS13* is 64.8%, while in the average, protein coding sequence it is only about 46% [72]. Additionally, ∼8.3% of all amino acids in WT ADAMTS13 are prolines (118 prolines), while prolines only make up 6.2% of amino acids in the human genome. Thus, although it is typical to perform these analyses using the complete human genomic codon frequencies, over-expression of ADAMTS13 in the transient transfection system enacts a significant change in codon usage within the cell. Therefore, both RSCU and the log ratio of codon frequencies were calculated not only using human genomic frequencies but also using *ADAMTS13* cDNA codon usages to mimic the strain of such over-expression. RSCU values were calculated for each variant using the variant codon. At all variant locations, the ΔRSCU value was computed between the variant and WT sequence.

### Log Ratio of Codon Frequencies

The frequency of the given codon divided by the frequency of all synonymous codons for the encoded amino acid was calculated for each variant. A log ratio of this frequency analysis was performed in order to assess differences between variant and WT. At each variant location, the log ratio of the frequency of the individual codon was calculated. Two different frequencies were used in these calculations: (i). Derived from human WT *ADAMTS13* cDNA codon frequencies (ii). Derived from the human Codon Usage Database [69]. For all calculations, a Δlog ratio of codon frequency value of variant versus the WT was calculated. A third method employed by Dos Reis *et al.* [73], was not used in the current study because the tRNA adaptation index does not take into account intercellular differences in tRNA content, which is necessary to address the relation of tRNA content to codon bias and translation speed.

### mFold and KineFold Analysis of mRNA Minimum Free Energy Structures

Using default parameters, short fragments of ADAMTS13 ORF (25, 75, 151, and 399 nt in length) were analyzed by mFold’s online server (http://mfold.rna.albany.edu/?q=mfold/RNA-Folding-Form) as well as the KineFold online server (http://kinefold.curie.fr/cgi-bin/form.pl); variants were centered within these sequences [24], [74]. To better understand the impact of variants on the minimum free energy of mRNA structure we have expanded the range of lengths to investigate. The most stable structure in terms of Gibbs free energy (ΔG) was determined and used for later analysis. Additionally, WT sequences of the same lengths were queried and a delta-delta G (ΔΔG) value was established by subtracting the Gibbs free energy value of WT from the variant. The ΔΔG was plotted and used for correlation studies.

*ADAMTS13 3-D Structure Prediction and Analysis*: Comparative modeling of the ADAMTS13 structure was performed using the 3D-PSSM algorithm developed by Kelley and Sternberg and Swiss-Pdb Viewer V4.0.2 using 1D and 3D sequence profiles coupled with secondary structure and solvation potential information. PSSM E-value (the score or “expectation value” of the match; “% Certainty”) was used to compare different model structures and the structures with the lowest E-value were chosen for further analysis [35]. Two model fragments/structures comprising ADAMTS13 residues 77–470 and 674–1254 (out of the 1427 total residues) were built with the help of PyMOL v0.98 and/or Swiss-Pdb Viewer V4.0.2 and the amino acids encoded by the codons/variants under investigation (whenever it was possible) were visualized (van der Waals’ radii of the side chains were shown). ADAMT13 fragment (comprising residues 287–682), whose structure was solved by X-Ray crystallography (PDB ID 3GHM) was also visualized using Swiss-Pdb Viewer V4.0.2 and the amino acids encoded by the codons/variants under investigation in this region (namely Gln448, Pro475, Arg484, Thr572, Pro618) were also visualized (van der Waals’ radii of the side chains were shown).

### Variant Conservation

Using the ConSeq server (http://conseq.tau.ac.il/ver1.1/index.html), the conservation of each individual variant was calculated [75], [76]. The conservation ranges from 1 (not conserved) to 9 (most conserved) and based on a multiple sequence alignment of 50 homologues proteins from the SwissProt database (http://www.uniprot.org/, last accessed 7 June 2011).

### Statistical Analysis

The mean *ADAMTS13* mRNA transcript level, intracellular & extracellular expression levels, and specific activity for the variants and the wild-type were compared using 2-sided *t* tests for independent samples, and a p-value less than.05 was considered to be of statistical significance. We performed correlation analysis using *ex vivo* and computational data. We first obtained computational data for variants and WT *ADAMTS13* mRNA/protein. The Spearman’s correlation coefficient between *in silico* and *ex vivo* data was then computed. Finally, we performed linear regression analysis for highly correlated cases where the correlation was statistically significant at or below 5% using ordinary least squares method. The statistical significance of ΔRSCU and Δlog ratio scores for our set of variants was evaluated by assigning a MAD score (robust Z-score) to each parameter. ΔRSCU and Δlog ratio for all variants within the coding region of ADAMTS13 were calculated based on data given the NCBI dbSNP database (http://www.ncbi.nlm.nih.gov/snp, last accessed 24 October 2011). Since the distribution of ΔRSCU and Δlog ratio for all variants within the coding region of ADAMTS13 cannot simply assumed to be normal, we used the MAD score as a more robust estimate of outliers. All ΔRSCU and Δlog ratio scores for our set of variants had |MAD score| <3 *i.e.* they fall within the range of median ±3MAD of the distribution of all variants within ADAMTS13.

Further, Spearman’s correlation matrix was computed for *in silico* data (correlation ≥0.6 or ≤−0.6; P<0.05) and *ex vivo* data in order to determine if highly correlating sets of computational data could possibly provide explanation for *ex vivo* data. We also looked at correlation of *ex vivo* data between themselves. Additionally, a linear regression analysis was performed for all the above cases to ensure that obvious outliers that would skew the correlation coefficient were not present.

## Supporting Information

Table S1 **Changes in predicted binding motif of splicing site regulators between variant and WT using SPmap web server.**
(DOC)

Table S2 **Summary of** ***in vitro*** **data for synonymous and nonsynonymous variants.**
(DOC)
