# Supplementary material for: Characterization of Coding Synonymous and Non-Synonymous Variants in ADAMTS13 Using Ex Vivo and In Silico Approaches
Source: PLoS One. 2012 Jun 29;7(6):e38864. doi: 10.1371/journal.pone.0038864 (PMC3387200; doi:10.1371/journal.pone.0038864)
Supplement: Table S1 — Changes in predicted binding motif of splicing site regulators between variant and WT using SPmap web server. (DOC) [file pone.0038864.s001.doc]

**Table S1: Changes in predicted binding motif of splicing site regulators between variant and WT using SPmap web server.**

| **Base Pair position** | **Amino Acid position** | **WT codon** | **Mutant Codon** | **WT/Variant** | **Motif** | **Splicing Factor** |
| --- | --- | --- | --- | --- | --- | --- |
| 354* | 118 | CCG | CCA | N/A |  |  |
| 420* | 140 | GCT | GCC | WT | ugcucc | MBNL |
|  |  |  |  | WT | ugcuc | CUG-BP |
| 1342 | 448 | CAA | GAA | Variant | gucgg | hnRNPH/F |
| 1423 | 475 | CCA | TCA | WT | uguacca | 9G8 |
|  |  |  |  | WT | guaccacac | YB1 |
| 1451 | 484 | AGA | AAA | WT | ugcagac | SF2/ASF |
|  |  |  |  | WT | ugcaga | SRp55 |
|  |  |  |  | WT | cagaca | hnRNPA1 |
|  |  |  |  | WT | ugcag | CUG-BP |
| 1716* | 572 | ACA | ACG | WT | ucac | NOVA1 |
|  |  |  |  | Variant | ugucacg | SRp40 |
| 1852 | 618 | CCC | GCC | WT | ccccucc | SRp20 |
| 2280* | 760 | GGT | GGC | N/A |  |  |
| 2699 | 900 | GTG | GCG | N/A |  |  |
| 2910* | 970 | GTC | GTT | Variant | agggguu | tra2beta |
| 3097 | 1033 | GCT | ACT | WT | ugcua | CUG-BP |
|  |  |  |  | Variant | acuac | SRp20 |
| 4221* | 1407 | ACC | ACA | N/A |  |  |

* Synonymous ADAMTS13 variants.
