# Supplementary material for: Characterization of Coding Synonymous and Non-Synonymous Variants in ADAMTS13 Using Ex Vivo and In Silico Approaches
Source: PLoS One. 2012 Jun 29;7(6):e38864. doi: 10.1371/journal.pone.0038864 (PMC3387200; doi:10.1371/journal.pone.0038864)
Supplement: Table S2 — Summary of in vitro data for synonymous and nonsynonymous variants. (DOC) [file pone.0038864.s002.doc]

**Table S2: Summary of *in vitro* data for synonymous and nonsynonymous variants.**

| **Base Pair position** | **Amino Acid position** | **mRNA Expression Level (Fold change as %WT)** | **Specific Activity (%WT)** | **Extracellular Protein Expression (%WT)** | **Intracellular Protein Expression (%WT)** |
| --- | --- | --- | --- | --- | --- |
| 354* | 118 | 0.99±0 | 207.2±77.3 | 137.1±16.8 | 146.2±21.2 |
| 420* | 140 | 1.09±0.26 | 68.6±28.1 | 119.7±26.5 | 130.2±52.5 |
| 1342 | 448 | 1.05±0.33 | 126.6±10.3 | 98.9±6.8 | 141.0±15.7 |
| 1423 | 475 | 0.53±0.7 | 57.5±5.3 | 102.7±10.3 | 130.5±6.8 |
| 1451 | 484 | 1.91±0.11 | 55.8±7.7 | 92.8±10.6 | 48.4±4.9 |
| 1716* | 572 | 0.75±0.04 | 94.6±21.7 | 109.9±11.2 | 81.8±6.7 |
| 1852 | 618 | 1.17±0.03 | 67.7±21.7 | 30.9±3.2 | 87.9±18.5 |
| 2280* | 760 | 1.38±0.15 | 180.8±40.5 | 77.1±10.3 | 140.3±37.6 |
| 2699 | 900 | 1.05±.02 | 72.5±25.9 | 86.0±11.2 | 28.8±13.2 |
| 2910* | 970 | 1.27±0.09 | 114.2±6.9 | 107.4±12.0 | 120.0±25.3 |
| 3097 | 1033 | 0.69±0.02 | 57.5±11.8 | 179.2±34.9 | 87.8±18.1 |
| 4221* | 1407 | 0.67±0.02 | 88.0±10.4 | 92.6±25.5 | 144.0±29.9 |

*Synonymous ADAMTS13 variants.
